# APOE4 astrocytes exhibit increased susceptibility to Aβ, tau, and cytokine‐induced mitochondrial dysfunction

**DOI:** 10.1002/alz70855_104761

**Published:** 2025-12-24

**Authors:** Siddhi S. Joshi, Josslen Thieschafer, Ling Li

**Affiliations:** ^1^ University of Minnesota, Minneapolis, MN, USA

## Abstract

**Background:**

APOE, produced by astrocytes in the brain, is encoded by three alleles—ε4, ε3, ε2. APOE undergoes lipidation to form high‐density lipoprotein like particles (HDL) that are crucial for cholesterol homeostasis, anti‐inflammation, and protein clearance. GWAS studies have identified apolipoprotein E4 (APOE4) as the greatest genetic risk factor for developing sporadic Alzheimer's disease (AD).

APOE isoforms exhibit differential effects on pathological hallmarks of AD including amyloid plaque load, neurofibrillary tangles and inflammatory responses (ε4>ε3>ε2). Moreover, bioenergetic alterations, mainly decreased glucose utilization, imbalanced mitochondrial flux and increased oxidative stress, have been implicated in AD. However, the impact of APOE4 on mitochondrial health and bioenergetic functions in AD is poorly understood. *We hypothesize that APOE isoforms differentially impact mitochondrial function with APOE4 increasing susceptibility to mitochondrial damage in AD*.

**Methods:**

Cellular models of astrocytes were used. Primary mouse astrocytes were derived from humanized APOE3 and APOE4 mice. Isogenic APOE3 and APOE4 human iPSC lines were differentiated into astrocytes using published protocols. Astrocytes were treated with synthetic amyloid‐β (Aβ) aggregates, tau isolates from transgenic PS19 mice of tauopathy, and cytokines to recapitulate accumulation of Aβ, neurofibrillary tangles and neuroinflammation *in vitro*. Rates of mitochondrial respiration, glycolysis and preference to key respiratory substrates were assessed in treated astrocytes using Seahorse flux analyzers. Mitochondrial membrane potential (MMP) was assessed using JC‐1 dye.

**Results:**

APOE4 disrupts mitochondrial bioenergetics by impairing mitochondrial respiration and over activating glycolysis in comparison to APOE3. APOE4 astrocytes also exhibit decreased MMP. Intriguingly, APOE4 astrocytes mainly rely on glucose as a fuel and are unable to utilize endogenous fatty acids for energy metabolism. Treatment with aggregated Aβ further exacerbates bioenergetic imbalance and decreases rate of ATP generation in APOE4 astrocytes in contrast to APOE3 astrocytes. Moreover, APOE genotype exerts differential effects on mitochondrial function in response to treatments with tau isolates and cytokines, respectively.

**Conclusion:**

This study provides experimental evidence of APOE4 linked mitochondrial dysfunction in astrocytes. APOE genotype differentially impacts mitochondrial health and bioenergetics in response to pathological stressors of AD. Future studies are warranted to assess the impact of astrocytic APOE4‐associated mitochondrial dysfunction on pathogenic processes of AD *in vivo*.

